# Neuro-Immunity Controls Obesity-Induced Pain

**DOI:** 10.3389/fnhum.2020.00181

**Published:** 2020-06-09

**Authors:** Tuany Eichwald, Sebastien Talbot

**Affiliations:** ^1^Département de Pharmacologie et Physiologie, Faculté de Médecine, Université de Montréal, Montreal, QC, Canada; ^2^Departamento de Bioquímica, Centro de Ciências Biológicas, Universidade Federal de Santa Catarina, Florianópolis, Brazil

**Keywords:** obesity, pain, white adipose tissue, ILC1, T_H_2, neuro-inflammation

## Abstract

The prevalence of obesity skyrocketed over the past decades to become a significant public health problem. Obesity is recognized as a low-grade inflammatory disease and is linked with several comorbidities such as diabetes, circulatory disease, common neurodegenerative diseases, as well as chronic pain. Adipocytes are a major neuroendocrine organ that continually, and systemically, releases pro-inflammatory factors. While the exact mechanisms driving obesity-induced pain remain poorly defined, nociceptor hypersensitivity may result from the systemic state of inflammation characteristic of obesity as well as weight surplus-induced mechanical stress. Obesity and pain also share various genetic mutations, lifestyle risk factors, and metabolic pathways. For instance, fat pads are often found hyper-innervated and rich in immune cell types of multiple origins. These immunocytes release cytokines, amplifying nociceptor function, which, in turn, via locally released neuropeptides, sustain immunocytes’ function. Here, we posit that along with mechanical stress stemming from extra weight, the local neuro-immune interplay occurring within the fat pads maintains the state of chronic low-grade inflammation and heightens sensory hypersensitivity. Overall, stopping such harmful neuro-immune crosstalk may constitute a novel pathway to prevent obesity-associated comorbidities, including neuronal hypersensitivity.

## Obesity

Obesity is defined as an excessive accumulation of fat associated with adverse health consequences and results from the complex interaction of socioeconomic, genetic, epigenetic, and lifestyle factors (WHO, 2018). Obesity prevalence has now reached epidemic proportions, affecting up to 1.9 billion adults worldwide, including 650 million patients who are clinically obese. The body mass index (BMI) is used to stratify adult patients as underweight (<18.5), normal weight (18.5–24.9), overweight (25–29.9), or obese (>30). The severity of obesity is further classified as class: I (30–34.9), II (35–39.9), or III (>40) (WHO, 2018).

In terms of mortality, it has been projected that obesity and obesity-associated chronic diseases will account for almost three-quarters of all deaths worldwide by 2020 (WHO, 2013). Thus, obesity is highly comorbid and constitutes a key risk factor for developing insulin resistance and type II diabetes, cardiovascular diseases, cancer, and dementia (Khaodhiar et al., 1999). In addition to these, obesity is now considered an important pain facilitator, as BMI and pain perception are strongly correlated (Table 1). For example, 40% of obese suffer from chronic pain, and the pain they report is more severe, often intractable, seeking more medical attention and consuming more painkillers (Thomazeau et al., 2014). Inversely, patients suffering from chronic pain are also more likely to become obese (Stone and Broderick, 2012). Being highly prone to develop osteoarthritis (OA), they chiefly report musculoskeletal pain (King et al., 2013). Finally, obesity-induced pain varies with the patients’ sex, diet, and body fat distribution.

**TABLE 1:** Pain sensitivity correlate with obesity status.

Patient sample	Pain site	Obesity prevalence among patients (%)	References
1 million adults	Diverse	44	Stone and Broderick, 2012
5,724 elderly	Knee	58	Andersen et al., 2003
721 adults	Headache	30	Tietjen et al., 2007
300 children and teenagers	Musculoskeletal	56	Taylor et al., 2006
182 adults	Diverse	40	Coaccioli et al., 2014
100 adult women	Fibromyalgia	45	Neumann et al., 2008

While significant advances were made in the understanding of the molecular mechanisms driving persistent pain, this knowledge has yet to translate into effective therapies. Thus, the management of chronic pain still constitutes a significant unmet clinical need (Yekkirala et al., 2017). The following section will explore the molecular mechanism and the relative contribution of (i) mechanical stress; (ii) chronic low-grade inflammation; and (iii) neuro-immunity to obesity-induced pain; and whether targeting such mechanism may constitute a therapeutic strategy to alleviate sensory hypersensitivity.

## Organization of the Nervous System and Pain Sensation

The nervous system is designed to quickly detect stimuli and direct avoidance behavior (Basbaum et al., 2009). In the periphery, autonomic neurons monitor and regulate organ functions (involuntary) while the somatic (voluntary) system provides sensitivity and motor control. In addition to these, nociceptors are a peripheral population of unmyelinated (C-fibers) or thinly myelinated (Aδ fibers) neurons specialized in sensing potentially damaging stimuli (pressure, temperature, chemical). These signals are transduced in electrical impulses, which are integrated centrally. In turn, effector signals are produced and transmitted to motor neurons activating muscle contraction (Albright et al., 2000) and occasionally tuning immune responses.

Upon activating nociceptor, the host generally experienced pain, which is defined as an unpleasant sensory and emotional experience (IASP, 2018). Pain is either acute, serving a protective physiological response to a harmful stimulus, or chronic, when physically debilitating and lasting over 3 months (IASP, 2018). Pain can also be stratified as (i) nociceptive, associated with the detection of potentially harmful tissue-damaging stimuli (Woolf and Ma, 2007); (ii) neuropathic, triggered by an injury/disease to the somatosensory nervous system (Costigan et al., 2009); (iii) dysfunctional, when associated to a disease state of the nociceptive nervous system (i.e., fibromyalgia) (Nagakura, 2015); or (iv) inflammatory, associated with tissue damage and active inflammation (Pinho-Ribeiro et al., 2017).

Nociceptors detect not just features typically thought of as causing a painful sensation, such as chemical and noxious temperature, but uncharacteristic signals ranging from cytokines, fungi, microbes, and immunoglobulins (Rajchgot et al., 2019). A revised portrait of neuron danger sensing include (i) ion channel transducers [transient receptor potential (TRP), Piezo]; (ii) receptor for sensitizing mediators [Mas-related G protein–coupled receptor (Mrgpr), prostaglandins (PG), protons (H), bradykinin (BK), etc.]; (iii) threat detectors including receptors for immunocyte-produced mediators (interleukins, chemokines, and immunoglobulins); and (iv) damage-associated molecular pattern (DAMP) detector (Crosson et al., 2019). Damage-associated molecular patterns, which include protease-activated receptors (PARs), toll-like receptors (TLRs), and pattern recognition receptors (PRRs), are families of specialized receptors for microbial proteins, and play an essential role in preserving mucosal homeostasis. Overall, nociceptors’ transcriptional data show the enormous variety of threat detection competences of nociceptor neurons, including immunocyte-produced cues (Wilson et al., 2013; Riol-Blanco et al., 2014; Talbot et al., 2015; Oetjen et al., 2017; Foster et al., 2017).

Inflammatory pain is characterized by the influx of immunocyte-producing cytokines, chemokines, and growth factors. These mediators typically bind to G protein-coupled receptors (GPCRs) and/or tyrosine kinase receptors expressed by nociceptor terminals and lead to intracellular kinases activation. Subsequently, these kinases decrease the activation threshold of ion channel transducer [i.e., transient receptor potential vanilloid-1 (TRPV1); transient receptor potential cation channel, subfamily A, member 1 (TRPA1)] and voltage-gated sodium channels (Na_V_) (i.e., Na_V_1.7, Na_V_1.8, and Na_V_1.9) (Caterina, 2000; Kerr et al., 2001; Nassar et al., 2004; Amaya et al., 2006; Bautista et al., 2006) or increased their membrane expression (Julius, 2013). Consequently, these effects sensitized nociceptors, which results in hypersensitivity to non-noxious stimuli, a situation termed allodynia (Woolf et al., 1992; Woolf and Ma, 2007). Besides, the nerve terminal “sensitized” state intensifies the response to painful trigger (termed hyperalgesia). Such hypersensitivity focuses the host on the injury site and leads to alternative behavioral (i.e., gait change). These effects are typically limited to the inflammatory sites, while systemic hypersensitivities result from central sensitization (Talbot and Couture, 2012; Rajchgot et al., 2019); see detailed review by Woolf et al. (1992) and Woolf and Ma (2007).

Known nociceptor sensitizer includes prostaglandin E_2_ (PGE_2_) produced by cyclooxygenase 2 (COX2) and nerve growth factor (NGF). Nociceptors express receptors for and were found to be sensitized by interleukin (IL)-1β (Binshtok et al., 2008) or Chemokine (C-C motif) ligand 3 (CCL3) (Zhang et al., 2005) in the context of pain, IL-5 in asthma (Talbot et al., 2015), IL-4 and thymic stromal lymphopoietin (TSLP) in atopic dermatitis (Oetjen et al., 2017), IL-31 during itch (Cevikbas and Steinhoff, 2012), IL-33 after contact with poison ivy (Liu et al., 2016), and IL-23 in the context of psoriasis (Riol-Blanco et al., 2014).

## Inflammation

Characterized by its pain, heat, redness, and edema (Larsen and Henson, 1983), inflammation suggests an immune-mediated phenomenon, but each of these characteristics can also stem from neuronal activation. Typically, noxious stimuli-induced action potential travels to the brain to initiate sensation (Talbot et al., 2016b; Azimi et al., 2017). When they reach the sensory neuron branch points, these electrical signals are also transmitted antidromically, back to the peripheral terminals, to initiate neurogenic inflammation via the local release of neuropeptides (Richardson and Vasko, 2002; Chiu et al., 2012). Subsequently, these peptides act on the endothelium to generate redness and heat (secondary to vasodilation) and edema (extravazation due to enhanced capillary permeability) (Foreman, 1987; Weidner et al., 2000).

## Immunity in Host Defense

Innate immunity is the primary barrier against microbes (Janeway and Medzhitov, 2002), while adaptive immunity generated long-term antigen memory (Litman et al., 2010). Antigen-presenting cells integrate danger signals and communicate information to T helper cells driving either of type 1 (against intracellular microbes), type 2 (against parasites), or type 17 (against extracellular pathogens) responses (Iwasaki and Medzhitov, 2015). Other immunocytes include mast cells (type 2 effector cells) (Metcalfe et al., 1997; Barbara et al., 2007), neutrophils (type 1 and 17 effector cells) (Mayadas et al., 2014), and macrophages (Hume, 2015). Macrophages have an incredible plasticity ranging from M1 macrophages (IL-1β^+^, TNF-α^+^), which are involved in cell elimination, to M2 macrophages (IL-10^+^, TGF-β^+^), partaking in tissue restoration. Besides, innate lymphoid cells (ILCs) are tissue-resident T_H_ cytokine-expressing cells (Lin^–^: TCRβ^+^, CD3ε^+^, CD19^+^, TCRγδ^+^, Ly6G^+^, F4/80^+^) (Spits et al., 2013), and as CD4 T cells, they are divided into three groups. ILC1 are T_H_1-like cells and produce INF-γ; ILC2 are T_H_2-like cells, express GATA3 and RORα, and produce IL-4, IL-5, and IL-13, while ILC3 are T_H_17-like, express RORγt, and secrete IL-17 and IL-22 (Artis and Spits, 2015). In the function of the cytokines they express, they guide response against pathogens, parasite, and fungi (Artis and Spits, 2015) and are also involved in autoimmune and inflammatory diseases (Fuchs and Colonna, 2013; Villanova et al., 2014).

## Mechanical Stress

Sustained overloading on the musculoskeletal structure of the lower back, hip, and knee joints prompts the development of OA in obese patients (King et al., 2013). Obesity-related OA is multifactorial and involves direct joint damage as well as genetic, biological, and metabolic factors (Davis et al., 1988). For instance, increased mechanical load changes the chondrocyte mechanotransducer signaling (Haudenschild et al., 2008), promotes cytokine secretion (IL-1β, IL-6, and TNF-α) and matrix metalloproteinases release, and contributes to establishing a pro-oxidative microenvironment (Stannus et al., 2010). These prompts the degradation of type II collagen, joint extracellular matrix, and hyaluronic acid fragmentation. These factors caused (i) imbalance between deterioration and repair of the cartilage; (ii) chondrocyte apoptosis; (iii) reduced synoviocyte fluid viscosity; and (iv) increased joint friction, all of which changed the patients’ posture and gait, reducing their mobility and increasing pain scores (Jordan et al., 2003).

While OA affects 16% of adults, it is present in 23% of overweight and 31% of obese patients (Vincent et al., 2012). As such, obese patients are 35% and 11% more at risk of developing knee and hip OA, respectively (Table 2; Jiang et al., 2012). Knee radiograph showed smaller width of the medial and lateral joint space in obese patients (Çimen et al., 2004), resulting in a 35% greater risk to undergo knee arthroplasty (Bourne et al., 2007).

**TABLE 2:** Obesity-mediated osteoarthritis (odds ratio).

Site	Obesity-related OR	References
Knee	1.6	Felson et al., 1988
Hip	2.0	Lievense et al., 2002
Hands	1.3	Haara et al., 2004
Low back	4.3	Liuke et al., 2005

These effects may be partly explained by the production of adipose tissue (AT)-secreted adipokines (leptin and adiponectin) (de Boer et al., 2012). These adipokines, whose circulating and cartilage levels correlate with BMI, activate their cognate receptors on the surface of chondrocytes, increasing the production of matrix metalloproteinases, nitric oxide (NO), and cytokines (IL-1β, IL-6, IL-8, TNF-α) (Figenschau et al., 2001). In turn, these mediators heighten synovial inflammation and pain hypersensitivity by driving fibroblast proliferation and immune cell infiltration (Francin et al., 2014).

## Obesity, a Low-Grade Inflammation Disease

White adipose tissue (WAT) comprised pre-adipocytes, adipocytes, endothelial cells, and immunocytes and has a primary role in energy storage. Adipocytes originate from mesenchymal stem cells (Qian et al., 2010), and their production is mainly controlled by epigenetic regulators, growth factors, and CCAAT-enhancer-binding proteins (or C/EBPs) and peroxisome proliferator-activated receptor γ (PPARγ) transcriptional regulators (Wu et al., 1999). Increased mitochondrial metabolism and biogenesis results in reactive oxygen species production, which initiate adipocyte differentiation in a mammalian target of rapamycin complex 1 (mTORC1)-dependent manner (Tormos et al., 2011). White adipose tissue content strikingly increases during obesity and, therefore, constitutes a predominant source of circulating hormones, peptides, cytokines, and adipokines (Hotamisligil, 2006).

It is increasingly recognized that immunological factors drive obesity induction, a phenomenon present in the WAT as well as pancreas, liver, and intestines (Osborn and Olefsky, 2012; Jin et al., 2013; Winer et al., 2016). Thus, lean AT is mainly composed of M2 macrophages, ILC2s, eosinophils, regulatory T cells (Tregs), and T_H_2 cells, while the obese fat pad is dominated by neutrophils, ILC1, M1 macrophages, and cytotoxic T cells. Similarly, the fat stromal vascular fraction of lean mice is composed of anti-inflammatory immune cells such as M2 macrophages (Lumeng et al., 2007a), Treg (Feuerer et al., 2009), and ILC2 (Molofsky et al., 2013). In contrast, M1 macrophages represent 10% and 40–50% of obese mice and patient stromal fractions, respectively (O’Rourke et al., 2012; Blaszczak et al., 2019). Such alternative and inflammatory composition support insulin resistance and maintain low-grade inflammation.

Adipocytes release the monocyte chemoattractant protein-1 (MCP-1), which attracts C-C chemokine receptor type 2 (CCR2)-expressing monocytes and favors their differentiation into M1 macrophages (Arner et al., 2012). Once in the WAT, macrophages form “crown-shaped structures” around dead adipocytes (Cinti et al., 2005), producing TNF-α, IL-1β, and IL-6 (Lumeng et al., 2007a). Therefore, M1 macrophages constitute one of the main source of cytokines in obese-WAT (Lumeng et al., 2007b) and, through PPARγ production, stimulate adipogenesis (Rosen et al., 2000). In addition, adipocytes tend to rupture due to their limited expansion capacity observed during obesity. The massive apoptosis of these cells drastically increased the levels of cytokines within the fat pad microenvironment and leads to the chemotaxis of M1 macrophages (Lumeng et al., 2007b).

Along with macrophages, the numbers of circulating monocyte and neutrophil increased during obesity (Poitou et al., 2011). Given that monocytes originate from hematopoietic stem cells (HSCs) and that obese patients have increased circulating HSCs progenitors, it was posited that HSCs might give rise to leukocyte influx (Bellows et al., 2011). Thus, HSCs enhance macrophage generation, via the myeloid differentiation primary response 88 (MyD88), a protein known to serve as an intermediate between extracellular danger signals sensed by TLR and the activation of the transcription factor nuclear kappa β (NF-κB) (Singer et al., 2014). These macrophages then go on to accumulate within the dorsal root ganglion (DRG) of high-fat diet (HFD)-fed mice (Song et al., 2017).

Diet-induced obesity CD4^+^ T lymphocytes were found to have biased T cell receptor (TCR) repertoires, suggesting an antigen-specific expansion. Glucose homeostasis typically becomes dysregulated in diet-induced obesity, when numbers of IFN-γ-secreting T_H_1 cells overwhelm the non-expending pools of T_H_2 (CD4^+^GATA-3^+^) and Tregs (CD4^+^CD25^+^Foxp3^+^). Through T_H_2 cell increases, CD4^+^ T cell transfer into HFD Rag1^–/–^ animals reversed weight increase and insulin resistance. Transient CD3 depletion also restores the T_H_1/Treg balance and reverses HFD-induced insulin resistance, suggesting an upstream role for CD4^+^ T cell in controlling obesity-associated metabolic abnormalities (Winer et al., 2009).

Adipose tissue macrophages (ATM) initiate WAT inflammation. However, recent data suggest that other tissue-resident innate immune cells, such as ILCs, also are major contributors (Yang et al., 2016). ILC2 number decreased in obese mice epididymis and human subcutaneous WAT (Brestoff et al., 2015). Being resident in lean AT, ILC2s maintain a T_H_2-like status of AT (Molofsky et al., 2013), in which ILC2-produced IL-5 and IL-13 promote the beiging of WAT (Molofsky et al., 2013; Brestoff et al., 2015; Hashiguchi et al., 2015). These effects are present in *Rag1* null mice and are, therefore, independent of tissue-resident M2 macrophages (Hams et al., 2013; Molofsky et al., 2013). Thus, IL-33-driven WAT biogenesis suggests another role for an ILC2-inducing cytokine in regulating obesity (Brestoff et al., 2015; Lee et al., 2015). In effect, IL-33 null mice gain more weight than their wild-type counterpart and have reduced frequency of ILC2s. This phenomenon is also present in IL-33 KO mice fed a normal diet (Brestoff et al., 2015). Exogenous IL-33 rescued WAT ILC2s number and M2 macrophage (Brestoff et al., 2015).

High-fat diet -exposed mice have enhanced IFN-γ levels (Wensveen et al., 2015). The depletion of natural killer (NK) cells decreased HFD-induced insulin resistance and M1 macrophage levels but stopped the onset of obesity. Inversely, the adoptive transfer of splenic NK cells into IFN-γ null animals restores HFD-mediated insulin resistance (Wensveen et al., 2015). Tissue-resident ILC1 may directly promote obesity-induced insulin resistance without the influence of natural killer T (NKT) or T cells (O’Sullivan et al., 2016). IL-12 activated ILC1 lead IFN-γ production and subsequent polarization of M1 macrophages (O’Sullivan et al., 2016). ILC1-derived IFN-γ balanced out the effect of IL-33 mediated ILC2 activation within visceral AT (Oboki et al., 2010), providing an *in situ* negative regulators of ILC2 anti-obesity effects.

While untested, ILC3-derived IL-17 may drive obesity-related comorbidities (Kim et al., 2014). Thus, Rag1 and IL-17, a double knockout mice, failed to develop asthma exacerbation when fed with a HFD (Kim et al., 2014). ILC3-producing IL-22 promote liver metabolism and help stop insulin resistance (Wang et al., 2014). Accordingly, *IL-22R* null mice were highly susceptible to HFD-induced obesity and insulin resistance. In obese mice, exogenous IL-22 tones down diabetes-induced oxidative stress and islet β inflammation, rescuing insulin secretion and glucose sensitivity (Hasnain et al., 2014).

Overall, the influx and polarization of immunocytes by WAT-derived mediators enhance fat accumulation, speed up joint damages, and may directly sensitize nociceptor, as discussed in the next section. Blocking the inflammatory component of obesity may, therefore, constitute a potential therapeutic avenue to stop obesity progression and its comorbidities.

## Innervation of the Wat

Under the control of a complex set of humoral and neural factors (Ismael et al., 2008; Dias et al., 2010; Talbot et al., 2012, 2016a; El Midaoui et al., 2015), WAT-released mediators tuned the host energy status as well as the number and phenotype of immune, vascular, and structural cells (Ouchi et al., 2011). Along with blood-derived factors, adipocyte size, lipid mobilization, and paracrine secretion are controlled by sensory nerve terminals (Bartness et al., 2014). Thus, fat pad sensory innervation is increased in obesity (Bamshad et al., 1998; Vaughan et al., 2014). In addition, evidences suggest a dual, yet segregated, sympathetic and parasympathetic innervation of WAT (Kreier et al., 2002). We refer the reader’s attention to the work of Bartness and colleagues for more information on WAT innervation (Bartness and Bamshad, 1998). Overall, neurons control WAT production of cytokines and immune influx, making fat innervation a central component player in obesity-induced low-grade inflammation.

## Autonomic Nervous System in Obesity

Sympathetic neurons control catabolic functions (Migliorini et al., 1997) via neuropeptide Y (NPY) suppression of lipolysis and promotion of angiogenesis and heighten adipocyte differentiation (Kuo et al., 2007). It is worth noting that adipocytes and macrophage-produced cytokines increase sympathetic flow, while excessive cytokine levels, such as during severe inflammation, have the opposite effect (Pongratz and Straub, 2014).

Parasympathetic vagal neurons that innervate the fat pad have anabolic functions helping tune insulin-mediated glucose and free fatty acid uptake and help promote lipid accumulation (Bartness, 2002). Conversely, lipid accumulation further increases their anabolic functions (Bartness, 2002). In doing so, norepinephrine controls triacylglycerol lipolysis, NO production, and tissue remodeling (Nguyen et al., 2018). Fat pad denervation decreased transcript expression of resistin and leptin, without impacting the levels of adiponectin. It, therefore, supports an anabolic role for WAT-parasympathetic neurons (Bartness, 2002; Kreier et al., 2002).

These data support the long appreciated contribution of parasympathetic neurons as an inhibitor of splenic macrophage-mediated inflammation (Tracey, 2002; Andersson and Tracey, 2012). Thus, spleen innervating adrenergic activates acetylcholinergic T cells (Rosas-Ballina et al., 2011). By stimulating splenic macrophages’ alpha-7 nicotinic receptor, acetylcholine stops their production of TNF-α (Wang et al., 2003).

Harnessing such inflammatory reflex using bioelectronic devices (Chavan et al., 2017), non-invasive vagus neurons activate blunt inflammation in arthritic patients (Koopman et al., 2016) and mice with experimental inflammatory bowel disease (Ji et al., 2014). As such, parasympathetic neuron activation constitutes an innovative approach to tone down systemic inflammation, obesity progression, and obesity-associated comorbidities, such as pain hypersensitivity.

## Neuro-Immunity

The sensory nervous and immune systems work together to promote host defense and homeostasis (Talbot et al., 2016b) and interact through a common language of receptors, cytokines, neuropeptides, and enzymes (Cronin et al., 2018). While this crosstalk is adaptive, protecting the host from threat, it drives disease pathophysiology (Chiu et al., 2013; Wilson et al., 2013; Talbot et al., 2015, 2016b; Foster et al., 2017). In addition to prompting withdrawal reflexes, neurons’ interaction with immunocytes controls host defenses (Barnes, 1996; Spiller, 2002; LaMotte et al., 2014; Talbot et al., 2016b).

Clinically, the denervation of an arthritic joint reversed inflammation in that joint, supporting a role for nociceptor regulation of inflammatory processes (Courtright and Kuzell, 1965). The somatosensory neurons are ideally positioned in lymphoid tissues and in the mucosal barrier to control immune responses (Talbot et al., 2015). Such microenvironment allows nociceptors to interact with tissue-resident immune cells via the local release of neuropeptides (Downing and Miyan, 2000; Rosas-Ballina et al., 2011; Foster et al., 2015; McMahon et al., 2015; Veiga-Fernandes and Mucida, 2016). The peptides generated and locally secreted during neurogenic inflammation promote lymphocyte polarization, controlling the extent and type (1, 2, or 3) of inflammation experienced (Ganea and Delgado, 2001; Goetzl et al., 2001; Cunin et al., 2011; Nussbaum et al., 2013; Talbot et al., 2015) as well as stimulating antigen trafficking in the lymph node (LN) (Valijan, 1989; Chiu et al., 2012; Roberson et al., 2013; Talbot et al., 2016b).

The effects of sensory neurons on immunity seem to vary between inflammatory context (T_H_1/T_H_17 vs T_H_2), the neuron subpopulation implicated, as well as the nature of the peptides being secreted (Foster et al., 2015; Azimi et al., 2016, 2017; Talbot et al., 2016b). Typically, substance P (SP) promotes T cell activity and increased dendritic cell (DC) recruitment and recognition of non-self-antigens (Calvo et al., 1992; Siebenhaar et al., 2007). Calcitonin gene-related peptide (CGRP) have the inverse action, stopping T cell proliferation and reducing DC migration to LN (Mikami et al., 2011).

During allergic airway inflammation, IL-5, a type II effector cytokine secreted from several immunocytes, directly activates airway nociceptors, and this leads to the release of VIP. In turn, VIP stimulates ILC2 (Nussbaum et al., 2013; Talbot et al., 2015) and CD4^+^ cells to induce cytokine production, including IL-5, which initiates a positive feedforward pro-inflammatory cycle (Talbot et al., 2015, 2016b; Foster et al., 2017). Neuromedin U (NMU) also stimulates ILC2s, and when given alongside IL-25, it enhances asthma severity (Cardoso et al., 2017; Klose et al., 2017; Wallrapp et al., 2017). Blocking the NMU–NMUR1 axis decreased ILC2 activity and allergic inflammation (Wallrapp et al., 2017). Besides, while CGRP supports ILC2 production of IL-5 (Wallrapp et al., 2019), it inhibits alarmin-driven type 2 cytokine production, constrains IL-13 expression, and blocks ILC2 proliferation (Nagashima et al., 2019; Wallrapp et al., 2019; Xu et al., 2019).

**FIGURE 1:**
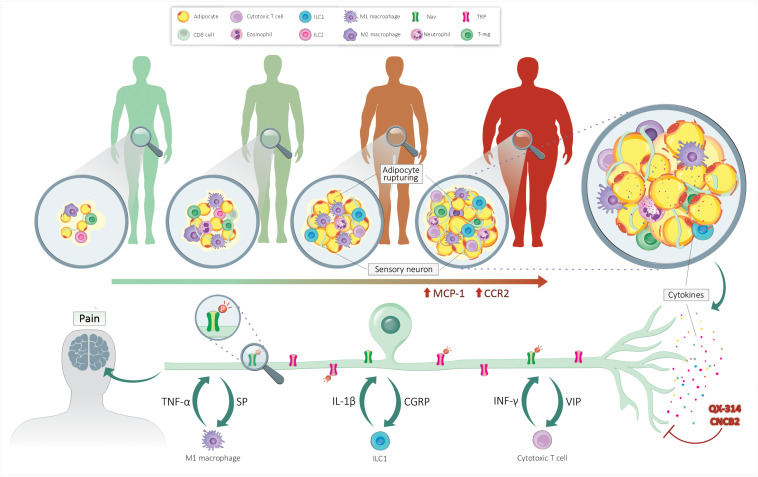
Neuro-immune crosstalk controls obesity-induced pain. Lean individual’s adipose tissue is sparsely innervated and comprises few adipocytes and anti-inflammatory immunocytes. The accumulation of fat leads to the rupture of adipocytes and the secretion of adipokines. These mediators increase the chemotaxis of immune cells, enhancing the level of pro-inflammatory cytokines. By acting on their cognate receptors present on sensory nerves, these cytokines sensitize nociceptor neurons by increasing the expression and phosphorylation of NaV and TRP channels. Upon sensitization, the sensory neurons secrete neuropeptides, further polarizing the fat pad’s immune cells. QX-314 silences nociceptor neuron’s electrical activity, while CNCB2 prevents their release of neuropeptides. By stopping neuro-immune crosstalk, these treatments would help resolve fat pad inflammation and blunt pain hypersensitivity.

Within the WAT microenvironment, locally released neuropeptides (CGRP, SP) increase immune influx and polarization, heightening WAT inflammation and nociceptor sensitivity (Foster and Bartness, 2006). From the data obtained in mouse models of allergic inflammation, one would imagine that CGRP produced by fat pad-innervating sensory neurons would block the function of ILC2s cells, unbalancing the type 1/type 2 immunocyte ratio within the WAT. By favoring T_H_1-mediated immunity, sensory neurons would enhance the influx of pro-inflammatory immune cells such as IL-1β and TNFα-secreting M1 macrophages and IFN-γ-producing ILC1. On the one hand, these cytokines would sensitize nociceptor neurons TRP and Na_V_ channels, triggering pain hypersensitivity; on the other hand, the neurons would locally release more neuropeptides to further imbalance the fat pad local immunity (hypothesized integrated system in Figure 1). Blocking the neuro-immune interplay in such a context would have a twofold, yet synergistic, effect: (i) directly decreased obesity-induced pain trigger by inflammatory cytokines, and (ii) decreased chronic low-grade inflammation. We devised two translational approaches to do this.

## Targeting Neuro-Immune Crosstalk

First, we modified an efficient pain and itch neuron-blocking strategy to locally silence tumor-innervating nociceptors (Roberson et al., 2013). This strategy uses large pore TRP channels as a specific drug delivery device to transport charged local anesthetic (such as QX-314) into nociceptor neurons to stop Na^+^ currents. In the context of inflammation, as found in the fat pad micro-environment, TRP channels open, allowing QX-314 (263 Da) to enter these neurons, producing a specific and durable electrical silencing (Binshtok et al., 2007). Of note, QX-314 did not impact immune cell function (Talbot et al., 2015), confirming its selectivity for inflammation-activated nociceptors (Talbot et al., 2015, 2020; Foster et al., 2017). This strategy offers three major potential advantages: (1) high specificity (the effect is limited only to sensory neurons that express activated large pore channels), (2) long-lasting activity, and (3) limited side effects; the charge on QX-314 would limit diffusion through lipid membranes and redistribution outside of the respiratory epithelium.

Second, Bean and colleagues devised novel charged N-type Ca^2+^ channel blockers, including an NCE termed CNCB2. The latter induced a prolonged pain blockade and was more potent than its neutral analog at inhibiting nociceptor release of CGRP and acted at lower concentrations to stop the neurogenic inflammation component of asthma. Such cationic molecules are therefore suited to treat pain by stopping potential action generation in nociceptive neurons and reducing inflammation by blocking pro-inflammatory neuropeptide release (Lee et al., 2019).

## Concluding Remarks

Peripheral sensitization is a major contributor to inflammatory pain (Albright et al., 2000; Woolf and Ma, 2007; Ji et al., 2014). Because several sensitizing mediators are released simultaneously during inflammation, stopping one of these mediators is likely to have a limited impact. Silencing sensitized neurons or shared downstream signaling pathways should therefore have larger and provide more prolonged pain relief. Among the others, QX-314, CNCB2, or activation of parasympathetic neurons using bioelectronic medicine, may constitute such broadly acting strategy in reversing the neuro-immune component of obesity-induced inflammation and pain.

## Author Contributions

All authors wrote the manuscript.

## Conflict of Interest

ST has an equity stake in Nocion Therapeutics. The remaining author declares that the research was conducted in the absence of any commercial or financial relationships that could be construed as a potential conflict of interest.
